# Molecular profiling of basal cell carcinoma of the prostate: A case report and literature review

**DOI:** 10.1016/j.eucr.2025.102993

**Published:** 2025-03-01

**Authors:** Luca Bertozzi, Eileen Zhang, Mohadese Behtaj, Olivia Gordon, Michael J. Whalen

**Affiliations:** a The George Washington University School of Medicine and Health Sciences, 2300 I St NW, Washington, DC, 20052, USA; b University of Maryland, 7901 Regents Drive, College Park, MD, 20742, USA; c Department of Pathology, The George Washington Medical Faculty Associates, 2300 M Street NW, Suite 715, Washington, DC, 20037, USA; d Department of Urology, The George Washington Medical Faculty Associates, 2150 Pennsylvania Ave NW, Suite 3-417, Washington, DC, 20037, USA

**Keywords:** Prostate cancer, Basal cell carcinoma, Molecular profiling

## Abstract

Prostate basal cell carcinoma (BCC) is a rare pathologic variant with a poorly understood molecular profile. Here, we describe a case of prostate BCC and compare its genetic alterations to cases in the literature. After presenting with hematuria, our patient underwent definitive radical prostatectomy for his localized biopsy-proven BCC. Somatic and germline testing revealed mutations in PIK3R1, KMT2D, and NOTCH1, and MUTYH, NBN, and MSH3, respectively. Upon literature review, we found that prostate BCC mutations disrupt cell growth, epigenetic regulation, and cell fate determination. With no consensus guidelines available, experimental targeted therapies have shown promise for prostate BCC management.

## Highlights

• Basal cell carcinoma (BCC) is a rare form of prostate cancer.
• Prostate-specific antigen may not be a reliable surveillance method for prostate BCC.
• Prostate BCC has genomic alterations that disrupt cell growth and differentiation.
• Targeted therapies may treat prostate BCC cases with actionable molecular targets.

## 1 Introduction

Prostate cancer is the most common solid malignancy in men,^1^ but BCC is a rarer histologic variant than the typical adenocarcinoma.^2^ Since reported cases of prostate BCC are limited, its malignant potential remains unclear. Given its heterogenous presentations, treatment approaches are also widely variable.^3^, ^4^, ^5^, ^6^, ^7^, ^8^, ^9^ Management options may include chemotherapy, radiation, radical prostatectomy (RP), androgen deprivation therapy (ADT), and/or targeted therapies, but there remain no consensus treatment recommendations for prostate BCC. With the advent of targeted therapies and emphasis on precision medicine in modern molecular oncology, molecular profiling of a patient's cancer can enable design of a personalized treatment regimen. Here, we offer a report on the clinical history of a patient with prostate BCC and a survey of its genomic profile.

## 2 Case presentation

**Fig. 1:**
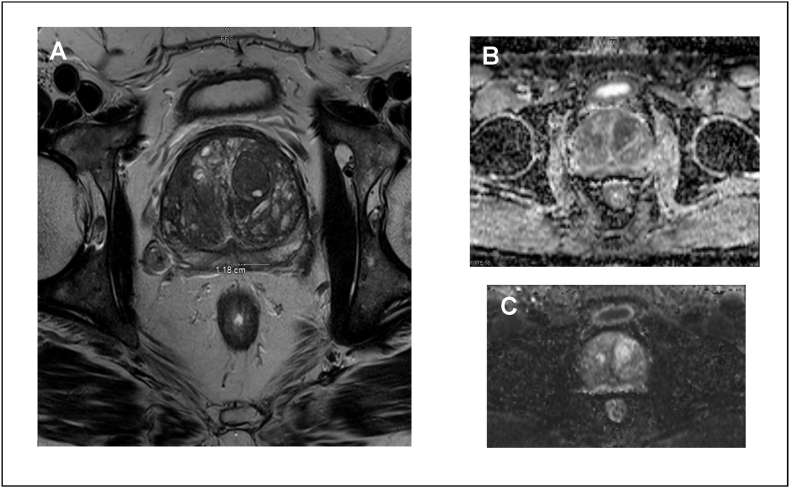
mpMRI of pelvis with and without contrast on 08/13/2021. **A** T2 axial view demonstrates homogenous low signal with a 1.1 × 1.5 cm left posterior peripheral zone, midgland lesion, PI-RADS 3. **B** Apparent diffusion coefficient map in axial view shows mild restricted diffusion. **C** High B-value diffusion weighted imaging in axial view shows mild restricted diffusion.

In January 2019, a 61-year-old man first presented with hematuria. The patient had a family history of prostate cancer of unknown pathology in his grandfather. Cystoscopy demonstrated benign prostate hyperplasia with trilobar hypertrophy and intravesical extension. He was monitored with serial prostate-specific antigen (PSA) measurements, and his PSA rose from 3.3 in November 2018 to 4.6 in November 2020. After this PSA increase, he underwent a transrectal ultrasound-guided prostate biopsy, which demonstrated atypical small acinar proliferation. Subsequent multiparametric magnetic resonance imaging (mpMRI) revealed a 1.1× 1.5-cm PI-RADS (Prostate Imaging Reporting and Data System) 3 lesion in the left posterior peripheral zone (Fig. 1A). Apparent diffusion coefficient and high B-value diffusion weighted imaging demonstrated mild restricted diffusion (Fig. 1B and C). PSA density based on volume of 136 cc was 0.03ng/mL/mL. Based on these radiographic findings and the atypia on original biopsy, the patient elected for repeat prostate biopsy, which demonstrated BCC. He initially postponed definitive treatment for social reasons and opted for cystolithalopaxy to treat his lower urinary tract symptoms secondary to cystolithiasis. With no indications of metastatic disease, radical prostatectomy was recommended for definitive treatment, which he underwent in December 2022.

**Fig. 2:**
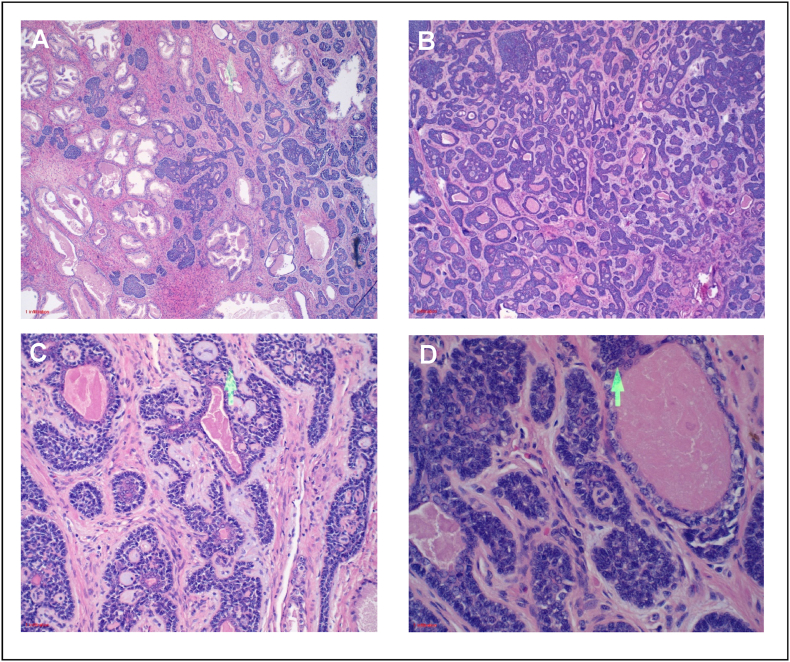
Basal cell carcinoma of prostate with adenoid cystic carcinoma (ACC)-like histologic patterns. **A** Variably sized nests of cells with peripheral palisading of basaloid cells and irregular anastomosing sheets of basaloid neoplastic cells, **B** with invasive pattern into the stroma and between normal prostate acini. **C** Cribriform architecture of the ACC-like pattern, **D** and central necrosis of the solid nests.

**Fig. 3:**
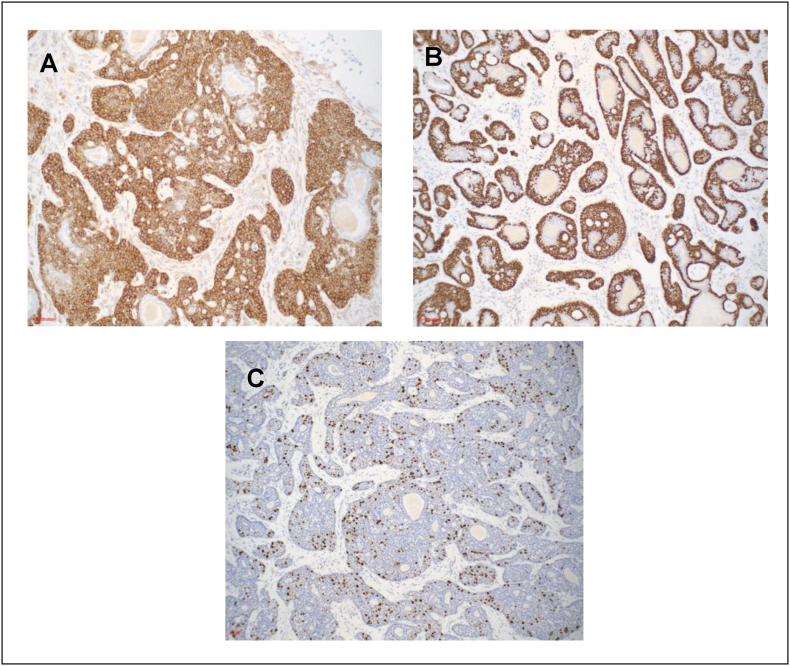
Immunohistochemical stains of basal cell carcinoma of prostate. **A** Tumor cells showing strong BCL2 positivity, **B** strong nuclear p63 immunoreactivity and **C** high Ki-67 proliferation index.

Final pathology was consistent with his original diagnosis of BCC, stage pT2N0MxR0. The tumor had variably sized nests of cells with peripheral palisading of basaloid cells, irregular anastomosing sheets of basaloid neoplastic cells, nests with prominent cribriform architecture (adenoid cystic carcinoma-like pattern) and cytoarchitectural atypia, infiltrating between normal prostate acini (Fig. 2A–D). The tumor cells were diffusely BCL-2+ and negative for PSA, HER-2, GATA-3, AMACR, chromogranin, and synaptophysin (Fig. 3A). High molecular weight cytokeratin and p63 highlighted the outermost layer and CK7 labeling adluminal layer within the tumor cells (Fig. 3B). Ki-67 proliferation index was high (>20 %) (Fig. 3C), potentially indicating a more aggressive malignancy.

To guide clinical decision-making, TEMPUS^10^ genetic testing panels were utilized on his prostate biopsy specimens. Somatic tissue testing (TEMPUS xT) revealed two frameshift mutations and one stop gain mutation in PIK3R1, a frameshift mutation in KMT2D, and a missense (G34C) mutation in NOTCH1. Tumor mutational burden was in the 19th percentile without microsatellite instability. Germline saliva testing (TEMPUS xG) revealed that the patient was heterozygous for MUTYH, NBN, and MSH3. Despite these insights, additional experimental targeted therapies were not indicated after prostatectomy due to the localized nature of his disease. Starting at 3 months after surgery, he has been undergoing surveillance with serial PSA measurements, although the optimal strategy for post-treatment surveillance in prostate BCC remains unclear.

## 3 Discussion

While serum PSA remains standard for long-term monitoring of most forms of prostate cancer, optimal surveillance strategies for prostate BCC are currently unknown. Exemplified by our patient, prostate BCC often stains negative for PSA, bringing into question the clinical utility of PSA for surveillance. As BCC often arises in the transition zone, studies have demonstrated limited associations of BCC with elevated PSA, concluding that prostate BCC may be best monitored with MRI, PSA density, or the 4K score.^5^^,^^6^^,^^11^ Radiographic surveillance with both computed tomography and MRI has been effectively employed in both localized^6^ and metastatic cases of prostate BCC.^4^^,^^12^ Until a more sensitive surveillance tool is developed, an effective observation plan for prostate BCC will depend on overall disease burden and key histologic features of the patient's tumor.

In these cases, Ki-67 staining had also been reported to be a reliable indicator of tumor proliferation. The presence of local invasion or necrosis on histology and an elevated Ki-67 index allows for distinction between malignant BCC and benign basal cell hyperplasia.^12^ While it is accepted that Ki-67 is a proliferation marker, other literature has speculated that high Ki-67 staining could indicate distant BCC metastasis.^12^ However, given the low incidence of BCC, more cases must be examined for a definitive answer. In prostate adenocarcinoma, the literature is more conclusive that Ki-67 correlates with malignant lesions and Gleason grade,^13^ a proven predictor of overall prognosis.

Next generation sequencing of malignant tissue informs us of the specific molecular mechanisms that drive oncogenesis, which is especially important when characterizing rarer neoplasms such as prostate BCC. Growth signaling aberrations, epigenetic dysregulation, and disruption of cell fate determination define the somatic mutational profile of our patient's tumor, which includes mutations in PIK3R1, KMT2D, and NOTCH1. PIK3R1 is a tumor suppressor gene involved in growth signaling pathways that is mutated in up to 6 % of prostate cancers.^4^ KMT2D is a component of a histone methyltransferase complex that has also been implicated as a putative driver gene in another prostate BCC case.^3^ Meanwhile, NOTCH1, classified as a “Variant of Uncertain Significance”, may be involved in cell fate determination, potentially suppressing tumorigenesis by impeding prostate cancer invasion.^14^

Examining germline changes can also help elucidate underlying molecular mechanisms and inheritance patterns of disease, which is critical when performing genetic counseling with patients. Our patient's germline mutations of MUTYH, NBN, and MSH3 may contribute to dysfunctional DNA damage repair mechanisms, potentially leading to oncogenesis. Although NBN and MSH3 are both considered “Variants of Uncertain Significance”, MUTYH is mutated in a form of hereditary polyposis that increases a patient's risk of genitourinary and gastrointestinal cancers.^15^ While much remains unknown behind the genetic mechanisms of prostate BCC development, germline mutation screening can help patients stratify risk within their families to optimize cancer prevention strategies.

Key prostate BCC mutations from the literature, in addition to the mutations in our patient, are summarized in Table 1. These genes have many functions but can be generally classified into three broad categories: chromatin remodeling, DNA damage repair, and cell development. One case of prostate BCC reported mutations in SMARCB and ATM.^4^ SMARCB regulates transcription through chromatin remodeling,^16^ and ATM is a DNA damage repair enzyme altered in up to 10 % of metastatic prostate cancer cases.^17^ MYB-NFIB, a fusion protein frequently implicated in prostate BCC according to fluorescence in situ hybridization analyses,^8^ consists of gene products that also regulate stem cells and promote chromatin accessibility. Another fusion protein identified in BCC is MSMB-NCOA4, which is associated with an increased risk of prostate cancer.^5^

**Table 1:** Table summarizing reported genomic alterations of prostate BCC. **Bold** indicates that the mutation was present in the patient described in our case report. RP = radical prostatectomy; SWI/SNF = switch/sucrose non-fermentable; RTK = receptor tyrosine kinase; NF-kB = nuclear factor kappa B; MAPK = mitogen-activated protein kinase.

Reported mutations of prostate BCC	Author, institution, and study type	Cases	Gene description	Mutation	Treatment
MYB-NFIB fusion	Magers et al., 2019, Indiana University School of Medicine, case series^8^	14	MYB: oncogenic transcription factor involved in regulation of stem and progenitor cells NFIB: oncogenic transcription factor that promotes chromatin accessibility	Translocation; somatic	N/A
ATM	Dong et al., 2020, Tianjin Medical University, case report^4^; Pedersen et al., 2021, Copenhagen University, case report^5^	2	DNA damage repair	Deletions; somatic + germline	^4^: Initial RP + radiation, then docetaxel + cisplatin, later etoposide for lung metastases ^5^: Initial transvesical prostatectomy, later pembrolizumab for abdominal wall metastases
**PIK3R1**	Current report; Dong et al., 2020, Tianjin Medical University, case report^4^	2	Oncogene that regulates the PI3K growth signaling pathway	Stop gain, point, deletions; somatic	Initial RP + radiation, then docetaxel + cisplatin, later etoposide for lung metastases
**KMT2D**/KMT2C	Current report; Su et al., Shanghai Jiao Tong University, case report^3^	2	Tumor suppressors that activate the PI3K/Akt pathway	Frameshift, in-frame deletion, missense; somatic	KMT2D: RP ^3^: docetaxel + prednisone + radiation
**NOTCH1**	Current report	1	Transmembrane receptor involved in cell fate determination and prostate cancer metastasis suppression	Missense; somatic	RP
**MUTYH**	Current report	1	DNA damage repair	Germline	RP
**NBN**	Current report	1	DNA damage repair	Germline	RP
**MSH3**	Current report	1	DNA mismatch repair	Polymorphism; germline	RP
CASC5	Su et al., Shanghai Jiao Tong University, case report^3^	1	Composes mitotic spindle assembly	Point; somatic	Docetaxel + prednisone + radiation
NUTM1	Su et al., Shanghai Jiao Tong University, case report^3^	1	Epigenetic modulator involved in male germ cell maturation	Point; somatic	Docetaxel + prednisone + radiation
PTPRC (CD45)	Su et al., Shanghai Jiao Tong University, case report^3^	1	Regulates B and T cell activation	Point; somatic	Docetaxel + prednisone + radiation
TBX3	Su et al., Shanghai Jiao Tong University, case report^3^	1	Regulates stem cell maintenance	Point; somatic	Docetaxel + prednisone + radiation
SMARCB1	Dong et al., 2020, Tianjin Medical University, case report^4^	1	Core subunit of SWI/SNF chromatin remodeling complexes	Deletions; somatic	Initial RP + radiation, then docetaxel + cisplatin, later etoposide for lung metastases
MSMB-NCOA4 fusion	Pedersen et al., 2021, Copenhagen University, case report^5^	1	MSMB: immunoglobulin secreted by prostate into semen NCOA4: oncogenic co-activator of androgen signaling pathway	Translocation; somatic	Initial transvesical prostatectomy, later pembrolizumab for abdominal wall metastases
FGFR2-TACC2 fusion	Rebhan et al., 2022, Medical University of Vienna, case report^9^	1	FGFR2: RTK that drives Ras/MAPK pathway in cell proliferation TACC2: stabilizes microtubules and interacts with chromatin remodelers	Translocation; somatic	Initial RP + staged extended lymphadenectomy, later pemigatinib for hepatic metastases
BRCA2	Grossman et al., 2018, Harvard Medical School, case report^7^	1	DNA damage repair	Polymorphism; germline	Initial olaparib, later atezolizumab and leuprolide acetate
CYLD	Low et al., 2022, Fred Hutchinson Cancer Center and Johns Hopkins University School of Medicine, primary research^18^	1	Tumor suppressor that deubiquitinates in NF-kB pathway	Stop gain; somatic	N/A

Modern genomics enables us to comprehensively characterize prostate BCC, which may ultimately inform its clinical course. Using whole genome sequencing, one study confirmed that the genomic alterations of prostate BCC are distinct from the changes seen in adenocarcinoma, and that both prostate adenocarcinoma and BCC have a low copy number and mutational burden.^18^ Copy number loss of chromosome 16 was also observed, as well as alterations in KIT, DENND3, PTPRU, MGA, and CYLD. High-throughput sequencing methods such as single-cell RNA-seq have enabled deep characterization of the prostate BCC genome, revealing key driver mutations of CASC5, NUTM1, PTPRC, KMT2C, and TBX3, genes also involved in chromatin remodeling and stem cell regulation.^3^ These authors retrospectively infer that the patient's cancer, which was radiosensitive but chemoresistant, may have responded better to radiation instead of targeted therapies since his tumor had fewer DNA damage repair gene mutations, exemplifying how molecular profiling can inform management strategies.

While unifying recommendations for prostate BCC therapy are yet to be formalized, some experimental targeted therapies have been reported in the literature. One patient with metastatic prostate BCC after RP was found to have an FGFR2-TACC2 chromosomal translocation and was treated with pemigatinib, an FGFR inhibitor.^9^ By targeting this fusion protein, whose gene products are involved in cell proliferation and chromatin remodeling, the patient experienced objective response with significant regression of his hepatic metastases. Another example of targeted therapy was reported in a patient with prostate BCC metastatic to the lung that underwent a BRCA2 reversion mutation.^7^ To target this germline BRCA2 mutation, olaparib, a PARP inhibitor that has demonstrated efficacy against metastatic prostate cancer,^19^ was initially administered and led to a complete clinical response after 3 months of treatment. After developing some resistance to olaparib, ADT and atezolizumab were briefly added to his regimen before resuming olaparib monotherapy. Notably, the patient's cancer continued to respond to olaparib even after developing the BRCA2 reversion mutation. This case illustrates the need to modify therapies in response to molecular changes arising from therapy-induced clonal selection pressure.

## 4 Conclusion

Modern targeted therapies demonstrate that treatment plans may be tailored to the unique molecular features of a patient's cancer that may be organ-agnostic. This treatment strategy may be useful for prostate BCC, a rare cancer with a relatively low mutational burden. Although a consensus genomic profile of prostate BCC is unavailable, somatic and germline testing can provide important data that may guide the application of targeted therapies. With unclear roles of chemotherapy, radiotherapy, and surgery in the management of prostate BCC, molecular findings may provide insights toward the rational treatment of advanced or metastatic disease, possibly leading to improvements in clinical outcomes for this rare form of prostate cancer.

## CRediT authorship contribution statement

**Luca Bertozzi:** Conceptualization, Formal analysis, Project administration, Writing – original draft, Writing – review & editing. **Eileen Zhang:** Visualization, Writing – original draft. **Mohadese Behtaj:** Data curation, Formal analysis, Visualization, Writing – original draft. **Olivia Gordon:** Writing – original draft. **Michael J. Whalen:** Data curation, Supervision, Writing – review & editing.

## Informed consent

Patient discussed in case presentation provided written informed consent to participate in the study.

## Data availability statement

Data sharing is not applicable to this article since no datasets were produced or analyzed in the current study. All data supporting this study are available in the paper.

## Funding

This research did not receive any specific grant from funding agencies in the public, commercial, or not-for-profit sectors.

## Declaration of competing interest

The authors declare that they have no known competing financial interests or personal relationships that could have appeared to influence the work reported in this paper.
